# Supplementary material for: AI alignment in medical imaging: Unveiling hidden biases through counterfactual analysis
Source: PLOS Digit Health. 2026 Aug 13;5(8):e0001516. doi: 10.1371/journal.pdig.0001516 (PMC13472419; doi:10.1371/journal.pdig.0001516)
Supplement: Supplementary file 1 [file pdig.0001516.s001.pdf]

Appendix

1 Missing proofs

1.1 Background on structural causal models

Definition 1 (Structural Causal Model (SCM), Definition 2.1 by [1])**.** *A structural causal model (SCM) is a tuple* $\langle \mathtt{I}, \mathtt{J}, \boldsymbol{V}, \boldsymbol{U}, \boldsymbol{f}, \mathbb{P}_{\boldsymbol{U}} \rangle$ *where (i)* $\mathtt{I}$ *is a finite index set of endogenous variables; (ii)* $\mathtt{J}$ *is a disjoint finite index set of exogenous variables; (iii)* $\boldsymbol{V} = \prod_{j \in \mathtt{I}} V_j$ *is the product of the domains of the endogenous variables, where each* V_j *is a standard measurable space; (iv)* $\boldsymbol{U} = \prod_{j \in \mathtt{J}} U_j$ *is the product of the domains of the exogenous variables, where each* U_j *is a standard measurable space; (v)* $\boldsymbol{f} \colon \boldsymbol{V} \times \boldsymbol{U} \to \boldsymbol{V}$ *is a measurable function that specifies the causal mechanism; (vi)* $\mathbb{P}_{\boldsymbol{U}} = \prod_{j \in \mathtt{J}} \mathbb{P}_{\boldsymbol{U}_j}$ *is a product measure, where* $\mathbb{P}_{\boldsymbol{U}_j}$ *is a probability measure on* $\boldsymbol{U}_j$ *for each* $j \in \mathtt{J}$*.*

In SCMs, the functional relationships between variables are expressed in terms of deterministic equations. This feature allows us to model the cause-effect relationships of the data-generating process (DGP) using *structural equations*. For a given SCM $\langle \mathtt{I}, \mathtt{J}, \boldsymbol{V}, \boldsymbol{U}, \boldsymbol{f}, \mathbb{P}_{\boldsymbol{U}} \rangle$ a structural equation specifies an endogenous random variable V_l via a measurable function of the form $V_l = f_{V_l}(\boldsymbol{V}, \boldsymbol{U})$ for all $l \in \mathtt{I}$. A *parent* $i \in \mathtt{I} \cup \mathtt{J}$ of l is any index for which there is no measurable function $g \colon \prod_{j \in \mathtt{I} \setminus \{i\}} V_j \times \boldsymbol{U} \to V_l$ with $f_{V_l} = g$ almost surely. Intuitively, each endogenous variables V_j is specified by its parents together with the exogenous variables, via the structural equations. A structural equations model as in Definition 1 can be conveniently described with the *causal graph*, a directed graph of the form $\mathcal{G} = (\mathtt{I} \cup \mathtt{J}, \mathcal{E})$. The nodes of the causal graph consist of the entire set of indices for the variables, and the edges are specified by the structural equations, i.e., $\{j \to l\} \in \mathcal{E}$ iff j is a parent of l. Note that the variables in the set $\mathsf{Pa}(V_l)$ are indexed by the parent nodes of l in the corresponding graph $\mathcal{G}$.

Interventions. We define the causal semantics of SCMs, by considering perfect interventions [2]. For a given a SCM as in Definition 1, consider a variable $\boldsymbol{W} \coloneqq \prod_{j \in \mathtt{I}'} V_j$ for a set $\mathtt{I}' \subseteq \mathtt{I}$, and let $\boldsymbol{w} \coloneqq \prod_{j \in \mathtt{I}'} v_j$ be a point of its domain. The perfect intervention $\boldsymbol{W} \leftarrow \boldsymbol{w}$ amounts to replacing the structural equations $V_j = f_{V_j}(\boldsymbol{V}, \boldsymbol{U})$ with the constant functions $V_j \equiv v_j$ for all $j \in \mathtt{I}'$. We denote with $V_l \mid do(\boldsymbol{w})$ the variable V_l after performing the intervention. This procedure define a new probability distribution $\mathbb{P}(v_l \mid do(\boldsymbol{W} = \boldsymbol{w}))$, which we refer to as interventional distribution. This distribution entails the following information: "Given that we have observed $\boldsymbol{W} = \boldsymbol{w}$, what would V_l have been had we set $do(\boldsymbol{W} = \boldsymbol{w})$, instead of the value $\boldsymbol{W}$ had actually taken?".

1.2 Proof of theorem 1

In order to prove Theorem 1, we use the well-known concept of valid adjustment set in causal inference. For a review on this notion and its properties we refer the reader to , e.g., [3, 4].

Definition 2 (Valid Adjustment Set)**.** *Let* $\mathcal{G}$ *be a causal graph and let* $A, \hat{Y}$ *be disjoint (sets of) nodes in* $\mathcal{G}$*. A set of nodes* $\boldsymbol{W}$ *is a valid adjustment set for* $\{A, \hat{Y}\}$*, if (i) no*

element in $\boldsymbol{W}$ is a descendant of any node which lies on a proper causal path from A to $\hat{Y}$, in the graph obtained by removing from $\mathcal{G}$ all incoming arrows into nodes in A; (ii) $\boldsymbol{W}$ blocks all non-causal paths from A to $\hat{Y}$ in $\mathcal{G}$.

Valid adjustment sets have the following important property: Let $\boldsymbol{W}$ be a valid adjustment set for $\{A, \hat{Y}\}$. Then, it holds $\mathbb{P}(\hat{Y} \mid do(A = a)) = \sum_w \mathbb{P}(\hat{Y} \mid A = a, \boldsymbol{W} = \boldsymbol{w})\mathbb{P}(\boldsymbol{W} = \boldsymbol{w})$, for all values a in the support of A. We now have all the tools to prove the following lemma.

Lemma 3. *Denote with $\hat{X}_a$ the random variable of a DCLDM and let $\hat{X}_A$ be the random variable obtained by evaluating the conditional family $\{\hat{X}_a\}_{a\in A}$ at the random variable A. Consider the predicted outcome $\hat{Y} = f(\hat{X}_A)$, for a classifier f. Then, it holds $\mathbb{P}(\hat{Y} \mid do(A = a), \boldsymbol{Z} = \boldsymbol{z}) = \mathbb{P}(\hat{Y} \mid A = a, \boldsymbol{Z} = \boldsymbol{z})$, for all possible values of A.*

Proof. Consider the generative process given by the DCLDM. In order to generate an image $\hat{x}_a$, we sample a representation vector $\boldsymbol{z}_T \sim \boldsymbol{Z}$ according to a Gaussian prior, and denoise this representation vector using a conditioning label $a \sim A$. Then, the predicted label of interest $\hat{Y}$ is given as $\hat{y} = f(\hat{x}_a)$. This generative process can be visualized with a causal graph as in Fig. 1. In this graph, the empty set is a valid adjustment set for $\{A, \hat{Y}\}$. Hence, the claim follows. □

We can now prove the main result, which we restate for completeness.

Theorem 1. *Consider a classifier f, e.g., a diagnostic model, with $\{c_1, \dots, c_n\}$ classes. Denote with $\{p_1(x), \dots, p_n(x)\}$ the probability distribution over classes for an input image x. For an input image x, denote with $f(\hat{x}_a)$ the prediction of f on a generated image with conditioning label $A = a$. Furthermore, define the quantity*

$$\mathsf{Avg}(\boldsymbol{z}, a) \coloneqq \sum_{i=1}^{C} c_i \cdot p_i(\hat{x}_a),$$

where $\hat{x}_a$ is sampled by denoising the representation $\boldsymbol{z}$ conditioned on a. Then, the model f is counterfactually invariant with respect to an attribute A if and only if it holds

$$\mathbb{E}\left[f(\hat{x}_a) \cdot \mathsf{Avg}(\boldsymbol{z}, a)\right] = \sum_{\hat{a}\in A} \mathbb{E}\left[f(\hat{x}_{\hat{a}}) \cdot \mathsf{Avg}(\boldsymbol{z}, \hat{a})\right] \tag{1}$$

In this formula, the expected values are taken for varying a and $\boldsymbol{z}$.

Proof. Following the notation of Lemma 3, note that by the conditional ignorability assumption we have that $\mathbb{E}[\hat{Y} \mid do(A = a), \boldsymbol{Z} = \boldsymbol{z}] = \mathbb{E}[\hat{Y} \mid A = a, \boldsymbol{Z} = \boldsymbol{z}]$ for all a. Hence, a model f is counterfactually invariant with respect to the attribute A if it holds $\mathbb{E}[\hat{Y} \mid A = a, \boldsymbol{Z} = \boldsymbol{z}] = \mathbb{E}[\hat{Y} \mid A = a', \boldsymbol{Z} = \boldsymbol{z}]$. Equivalently, a model is counterfactually invariant if it holds

$$\mathbb{E}\left[\left(\mathbb{E}[\hat{Y} \mid A, \boldsymbol{Z}] - \mathbb{E}[\hat{Y} \mid \boldsymbol{Z}]\right)^2\right] = 0,$$

where the outer expected value is taken on A and $\boldsymbol{Z}$. By the tower property of the expectation [5], we have that

$$\begin{aligned}
\mathbb{E}\left[\left(\mathbb{E}[\hat{Y} \mid A, \boldsymbol{Z}] - \mathbb{E}[\hat{Y} \mid \boldsymbol{Z}]\right)^2\right] &= \mathbb{E}\left[\mathbb{E}\left[\left(\mathbb{E}[\hat{Y} \mid A, \boldsymbol{Z}] - \mathbb{E}[\hat{Y} \mid \boldsymbol{Z}]\right)^2 \mid \boldsymbol{Z}\right]\right] \\
&= \mathbb{E}\left[\mathbb{E}\left[(\mathbb{E}[\hat{Y} \mid A, \boldsymbol{Z}]^2 - \mathbb{E}[\hat{Y} \mid A, \boldsymbol{Z}]\mathbb{E}[Y \mid \boldsymbol{Z}]) \mid \boldsymbol{Z}\right]\right] \\
&= \mathbb{E}\left[\mathbb{E}[\hat{Y} \mid A, \boldsymbol{Z}]^2\right] - \mathbb{E}\left[\mathbb{E}\left[\mathbb{E}[\hat{Y} \mid A, \boldsymbol{Z}]\mathbb{E}[\hat{Y} \mid \boldsymbol{Z}] \mid \boldsymbol{Z}\right]\right] \\
&= \mathbb{E}\left[\mathbb{E}[\hat{Y} \mid A, \boldsymbol{Z}]^2\right] - \mathbb{E}\left[\mathbb{E}[\hat{Y} \mid \boldsymbol{Z}]^2\right] \\
&= \mathbb{E}\left[\hat{Y}\mathbb{E}[\hat{Y} \mid A, \boldsymbol{Z}]\right] - \mathbb{E}\left[\hat{Y}\mathbb{E}[\hat{Y} \mid \boldsymbol{Z}]\right].
\end{aligned}$$

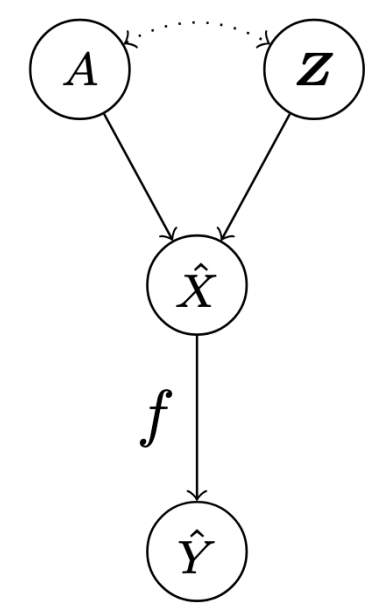

Fig 1. Causal structure for the DGP induced by the DCLDM.

The claim follows, since for a classifier f with $\{c_1, \dots, c_n\}$ classes and a discrete random variable A, we have that f is counterfactually invariant if and only if it holds

$$\begin{aligned}\mathbb{E}\left[f(\hat{x}_a) \cdot \mathsf{Avg}(\boldsymbol{z}, a)\right] &= \mathbb{E}\left[\hat{Y}\mathbb{E}[\hat{Y} \mid A, \boldsymbol{Z}]\right] \\ &= \mathbb{E}\left[\hat{Y}\mathbb{E}[\hat{Y} \mid \boldsymbol{Z}]\right] \\ &= \sum_{\hat{a}\in A} \mathbb{E}\left[f(\hat{x}_{\hat{a}}) \cdot \mathsf{Avg}(\boldsymbol{z}, \hat{a})\right],\end{aligned}$$

as claimed. □

2 Datasets description

2.1 Synthetic datasets

We consider six synthetic datasets, namely, linear, quadratic, exponential, interactive, log-exponent, and sin. Each synthetic datasets consist of features $\boldsymbol{Z} = \{Z_0, \dots, Z_n\}$ together with a protected feature A, as well as observations X and corresponding labels Y. The DGP is described by the following equations:

$$\begin{cases} A & \sim \textsc{Bernoulli}(0.3) \\ Z_i & = M_i Z_{i+1} A + N_i Z_{i+1}(1-A) + \xi_i, \ t = 0, 1, .., n-1 \\ X & = M_X Z_0 + r + \epsilon_X \\ Y & \sim \textsc{Bernoulli}(\textsc{Sigmoid}(f_Y(Z_n))) \end{cases}$$

In this data generating process, parameters $M_i, N_i, M_X \in \mathbb{R}^{k\times k}$ are transformation matrices; $r \in \mathbb{R}^k$ is a parameter representing the bias; $\xi_i, \epsilon_X \sim \mathcal{N}(0, \sigma^2)$ are random errors. f_Y is a transformation layer that adds nonlinearity before the sigmoid activation. For each transformation, we use a different random realization of the parameters, thereby generating different synthetic datasets. We apply one linear transformation and five nonlinear transformations for f_Y:

- linear: $f_Y(Z_n) = \omega_Y \cdot Z_n + b_Y$, $\omega_Y \sim \mathcal{N}(\mathbf{0}, I)$, $b_Y \sim \mathcal{N}(0, 1)$.
- quadratic: $f_Y(Z_n) = \omega_Y \cdot (Z_n)^2 + b_Y$, $\omega_Y \sim \mathcal{N}(\mathbf{0}, 4I)$, $b_Y \sim \mathcal{N}(20, 1)$.
- exponential: $f_Y(Z_n) = \omega_Y \cdot e^{Z_n} + b_Y$, $\omega_Y \sim \mathcal{N}(\mathbf{0}, I)$, $b_Y \sim \mathcal{N}(10, 1)$.
- interactive: $f_Y(Z_n) = \sum_{i\neq j} \omega_Y^{i,j} \cdot Z_n^i \cdot Z_n^j + b_Y$, $\omega_Y^{i,j} \sim \mathcal{N}(0, 1)$, $b_Y \sim \mathcal{N}(0, 1)$.
- log-exponent: $f_Y(Z_n) = \log(\exp(\omega_Y \cdot Z_n) + b_Y)$, $\omega_Y \sim \mathcal{N}(0, I)$, $b_Y \sim \mathcal{N}(5, 1)$.

- sin: $f_Y(Z_n) = \omega_Y \cdot \sin(Z_n) + b_Y$, $\omega_Y \sim \mathcal{N}(\mathbf{0}, I)$, $b_Y \sim \mathcal{N}(2, 1)$.

Here, ω_Y and b_Y are fixed model parameters during the generation process. During the simulation, we take the following values on the hyperparameters $T = 3, k = 32, \sigma = 0.01$. The SCM's parameter M_t, N_t are random matrix whose elements are sampled from uniform distribution $[-10, 10]$. The initial state $Z_n \sim \mathcal{N}(0, I_k)$. For reproducibility, we publish the synthetic data on Zenodo (https://doi.org/10.5281/zenodo.18803863).

2.2 The CheXpert dataset

CheXpert is a large-scale dataset comprising $224,316$ chest radiographs from $65,240$ patients [6]. Each image is annotated for the presence of common chest radiographic observations. Following previous work [7], we focus on five of these observations in our experiments: Cardiomelagy, Edema, Consolidation, Atelectasis, and Pleural Effusion. The CheXpert dataset also provides demographic labels, including self-reported race and sex. Overall, 55.5% of patients are labeled as "male" and 44.5% as "female". Regarding race, 4.8% of patients are labeled as "black", 10.8% as "asian", 56.2% as "white", and 28.2% as "unknown". For our experiments, we filter the CheXpert dataset to include only frontal chest images and restrict the racial categories to "black", "white", and "asian".

2.3 The MIMIC-CXR dataset

MIMIC-CXR [8, 9] is a large, publicly available dataset comprising chest X-ray images and their corresponding radiological reports. Similar to the CheXpert dataset, image labels are automatically extracted from the reports, and we focus on 5 out of the 14 available observations: Cardiomegaly, Edema, Consolidation, Atelectasis, and Pleural Effusion. The dataset also includes patient demographic information, such as sex and self-reported race, providing a research setting consistent with that of CheXpert. In this dataset, 52.17% of patients are labeled as "male" and 47.83% as "female". Regarding race, 3.2% of patients are labeled as "asian", 18.5% as "black", and 67.6% as "white". For our experiments, we restrict the dataset to patients labeled as "black", "white", or "asian", and include only frontal-view images.

3 Base classifiers for experimental comparison

3.1 Base classifiers for the synthetic datasets

In our experiment the examined objects are classifiers that predicts Y given X. We create a diverse model pool containing 1000 statistical learning models. To achieve this, we first define several base model architectures, such as Support Vector Classifiers, Logistic Regression, Decision Trees, and ensemble methods. For each of the base architectures, we establish a comprehensive hyperparameter configuration, which is shown in Table 1. This process results in a large set of unique ML model configurations (over 400 distinct combinations). The final pool of 1000 instantiated models is then created by sampling from these configurations and setting random seeds. The implementation is directly sourced from the scikit-learn package.

3.2 Base classifiers for CheXpert and MIMIC-CXR

Model architectures. Same setting is applied to CheXpert and MIMIC-CXR dataset. The diagnosis models, namely the disease classifiers, are trained to predict five diseases simultaneously. The five diseases are cardiomegaly, edema, consolidation and

Table 1. Machine learning models and their hyperparameters used for classification on the synthetic datasets.

Model	Hyperparameter Search Space
Support Vector Classifier (SVC)	For Linear Kernel (‘kernel=’linear’‘):<br>$C \in \{0.01, 0.1, 1, 10, 100\}$<br>*(Total Combinations: 5)*<br><br>For RBF Kernel (‘kernel=’rbf’‘):<br>$C \in \{0.1, 1, 10, 100\}$<br>$\gamma \in \{0.001, 0.01, 0.1, 1, \text{'scale'}\}$<br>*(Total Combinations: 20)*<br><br>For Polynomial Kernel (‘kernel=’poly’‘):<br>$C \in \{0.1, 1, 10, 100\}$<br>degree $\in \{2, 3, 4\}$<br>$\gamma \in$ {’scale’, ’auto’}<br>*(Total Combinations: 24)*
Logistic Regression	$C \in \{0.01, 0.1, 1, 10, 100\}$<br>penalty ∈ {’l1’, ’l2’}<br>solver ∈ {’liblinear’, ’saga’}<br>*(Total Combinations: 20)*
Decision Tree Classifier	criterion ∈ {’gini’, ’entropy’}<br>max_depth $\in \{5, 10, 20, 30, \text{None}\}$<br>min_samples_split $\in \{2, 5, 10\}$<br>min_samples_leaf $\in \{1, 2, 4\}$<br>*(Total Combinations: 90)*
Random Forest Classifier	n_estimators $\in \{50, 100, 200\}$<br>max_features ∈ {’sqrt’, ’log2’}<br>max_depth $\in \{10, 20, \text{None}\}$<br>min_samples_split $\in \{2, 5\}$<br>min_samples_leaf $\in \{1, 2\}$<br>*(Total Combinations: 72)*
Gradient Boosting Classifier	n_estimators $\in \{50, 100, 200\}$<br>learning_rate $\in \{0.01, 0.1, 0.2\}$<br>max_depth $\in \{3, 5, 8\}$<br>subsample $\in \{0.7, 0.8, 1.0\}$<br>*(Total Combinations: 81)*
MLP Classifier	hidden_layer_sizes $\in \{(16, 4), (16, 8, 4),$<br>$(32, 16), (50,), (100, 50)\}$<br>activation ∈ {’relu’, ’tanh’}<br>solver ∈ {’adam’, ’sgd’}<br>alpha $\in \{0.0001, 0.001, 0.01\}$<br>*(Total Combinations: 90)*

pleural effusion. All the models have the same high level structure: the image is processed through a backbone to produce high-level feature maps. The feature map then goes through a global average pooling operation, reducing the spatial dimension to $(1, 1)$. Then for each of the five labels, dropout and batch normalization are applied before passing through a label-spECAfic 1×1 convolutional classifier to obtain the logits. We consider three types of CNN models, namely Densenet[10], VGG [11] and inception-V3[12]. For each type of backbone we select multiple variations. For Densenet, we select Densenet 121, Densenet 169 and Densenet 201. For VGG, we select VGG13, VGG13+BN, VGG16, VGG16+BN, VGG19, VGG19+BN. For VGG family's models, '+BN' means that an additional batch normalization is applied in each convolution layer.

Training details. The training, validation and test set are splitted in the same way as we did in training the DCLDM for CheXpert and MIMIC-CXR respectively. Images are resized to 256×256 with the pixel value scaled to $[-1, 1]$. For each model, we download the pretrained backbone from torchvision and then train the full model with a batchsize of 64 and a learning rate of $1e^{-4}$ for 20 epochs. The loss is computed as the

Table 2. ROC-AUC Scores of 10 classification models for five diseases across demographic groups on the CheXpert dataset

Model	Race	Cardiomegaly	Edema	Consolidation	Atelectasis	Pleural Effusion
densenet121	Black	0.820	0.710	0.961	0.770	0.830
	White	0.880	0.780	0.943	0.720	0.870
	Asian	0.810	0.730	0.925	0.770	0.850
densenet169	Black	0.870	0.870	0.950	0.830	0.850
	White	0.953	0.872	0.941	0.810	0.830
	Asian	0.892	0.880	0.931	0.840	0.850
densenet201	Black	0.840	0.850	0.944	0.800	0.900
	White	0.940	0.871	0.950	0.800	0.870
	Asian	0.881	0.884	0.926	0.820	0.860
VGG13	Black	0.860	0.850	0.961	0.830	0.890
	White	0.930	0.900	0.930	0.750	0.870
	Asian	0.880	0.949	0.918	0.790	0.830
VGG13+BN	Black	0.850	0.830	0.963	0.810	0.900
	White	0.890	0.820	0.931	0.810	0.910
	Asian	0.860	0.830	0.926	0.850	0.930
VGG16	Black	0.830	0.860	0.964	0.790	0.900
	White	0.890	0.850	0.960	0.800	0.860
	Asian	0.860	0.870	0.926	0.760	0.850
VGG16+BN	Black	0.820	0.870	0.950	0.840	0.880
	White	0.900	0.920	0.921	0.820	0.860
	Asian	0.870	0.910	0.911	0.840	0.870
VGG19	Black	0.870	0.850	0.971	0.840	0.830
	White	0.940	0.910	0.930	0.810	0.810
	Asian	0.890	0.920	0.930	0.850	0.830
VGG19+BN	Black	0.840	0.870	0.951	0.810	0.900
	White	0.900	0.920	0.940	0.810	0.870
	Asian	0.870	0.920	0.928	0.800	0.830
Inception V3	Black	0.830	0.850	0.931	0.840	0.910
	White	0.890	0.860	0.940	0.790	0.870
	Asian	0.850	0.891	0.926	0.820	0.840

sum of binary cross entropy loss of five labels. We use Adam optimizer.
Performance across demographic groups. We report the performance of the diagnostic models by describing the ROC-AUC scores. Results are shown in Table 2 (CheXpert) and Table 3 (MIMIC-CXR).

4 Implementation details

4.1 Conditional Latent Diffusion Models

Model architecture. The DCLDM consists of two components, the outside structure VAE and the inner structure diffusion model. The encoder is constructed as a series of convolutional layers and downsampling modules, which progressively reduce the spatial dimensions while capturing essential features from the input data. The decoder, on the other hand, consists of convolutional layers and upsampling modules, designed to reconstruct the original data from the latent representation. To enhance the model's performance, attention mechanisms and skip connections are employed. The DDPM uses U-net as the noise estimator $\epsilon_\theta(\boldsymbol{z}_t, t, a)$ to denoise the latent space. The U-net is further augmented by a cross-attention mechanism to enable layer propagation with conditioned label. Technically, we adopt the classic archilatent diffusion tecture used for medical images[13].

Training details. The outside VAE structure and the diffusion model inner structure are trained in two successive phases. In the first phase, the VAE is trained to encode a $256 \times 256 \times 1$ image into a $32 \times 32 \times 8$ latent representation and then decode it back into the original image space. Here the number of channels of the latent code adopts the strategy in [14] which shows good performance. A multi-resolution loss function,

Table 3. ROC-AUC Scores of 10 classification models for five diseases across demographic groups on MIMIC-CXR

Model	Race	Cardiomegaly	Edema	Consolidation	Atelectasis	Pleural Effusion
densenet121	Black	0.792	0.747	0.962	0.825	0.853
	White	0.854	0.819	0.944	0.775	0.887
	Asian	0.780	0.754	0.928	0.830	0.864
densenet169	Black	0.844	0.915	0.949	0.875	0.873
	White	0.926	0.915	0.939	0.862	0.857
	Asian	0.860	0.916	0.927	0.900	0.875
densenet201	Black	0.820	0.896	0.944	0.852	0.918
	White	0.912	0.913	0.951	0.861	0.898
	Asian	0.850	0.930	0.926	0.875	0.875
VGG13	Black	0.832	0.892	0.960	0.872	0.905
	White	0.898	0.946	0.931	0.810	0.898
	Asian	0.852	0.990	0.917	0.854	0.863
VGG13+BN	Black	0.821	0.872	0.964	0.865	0.916
	White	0.857	0.867	0.930	0.869	0.943
	Asian	0.831	0.871	0.926	0.898	0.968
VGG16	Black	0.806	0.896	0.965	0.851	0.920
	White	0.853	0.888	0.960	0.865	0.897
	Asian	0.826	0.905	0.928	0.822	0.868
VGG16+BN	Black	0.792	0.914	0.949	0.887	0.897
	White	0.872	0.966	0.920	0.887	0.897
	Asian	0.847	0.952	0.913	0.877	0.892
VGG19	Black	0.841	0.898	0.970	0.887	0.858
	White	0.912	0.957	0.932	0.862	0.856
	Asian	0.861	0.967	0.930	0.896	0.854
VGG19+BN	Black	0.818	0.911	0.949	0.857	0.916
	White	0.871	0.960	0.939	0.864	0.902
	Asian	0.841	0.961	0.930	0.877	0.857 height
Black	0.806	0.894	0.930	0.908	0.931	Inception V3
	White	0.861	0.902	0.941	0.854	0.911
	Asian	0.817	0.926	0.927	0.870	0.867

combined with KL-regularization, is used as the training objective. In the second phase, the VAE is frozen, and the input image is encoded into the latent space by the encoder. Random noise is then sampled and used to diffuse the noised latent vector over 1000 steps. The U-Net [15] is subsequently trained to predict the noise from the diffused sample, following the standard training procedure of the diffusion model. During sampling, the diffusion model strategy [16] is applied to accelerate the iterative process.

Variance control. For diffusion models, the hyperparameter β_t, $\alpha_t = 1 - \beta_t$ and the number of steps T controls the noise's variance during the forward process, where data is gradually corrupted by adding Gaussian noise. β_t governs how much noise is added. In settings where the goal is to generate diverse images, the number of steps and β_t are usually set to 1000 and a linear interpolation between 0.001 to 0.01. In the counterfactual image generation, however, large β and T could lead to excessive variance in the generated images. To overcome this problem, in our analysis we use a variance reduction technique. Empirical studies [17] show that steps between 50 to 500 can still produce high-quality images with reduced variance. Therefore in our task, we actively reduce β to 20% and T to 250 to fit our counterfactual demand. Experiments show that diffusion models achieves lower sample variance without significantly compromising the quality of the generated images.

4.2 Learning the disentangling transformation

To improve performance, we remove the influence of the original sensitive attribute (e.g., self-reported race) from the patient's latent representation $\boldsymbol{z}_0$. We achieve this by learning a non-linear transformation $\phi_{\lambda,\delta}$ that makes the resulting representation $\tilde{\boldsymbol{z}}_0 = \phi_{\lambda,\delta}(\boldsymbol{z}_0)$ disentangled from the observed attribute A.

The optimal transformation is one that minimizes the Mutual Information (MI)

Table 4. Network architecture for m_δ.

Layer/Block	Module	Output Shape
**Input**	Input Tensor	(32, 32, 8)
**Conv Block 1**	Conv2D(16, 3x3) + ReLU<br>BatchNorm<br>Conv2D(16, 3x3) + ReLU<br>BatchNorm<br>MaxPooling(2x2)	(16, 16, 16)
**Conv Block 2**	Conv2D(32, 3x3) + ReLU<br>BatchNorm<br>Conv2D(32, 3x3) + ReLU<br>BatchNorm<br>MaxPooling(2x2)	(8, 8, 32)
**Conv Block 3**	Conv2D(64, 3x3) + ReLU<br>BatchNorm<br>Conv2D(64, 3x3) + ReLU<br>BatchNorm<br>MaxPooling(2x2)	(4, 4, 64)
**Upsample Block 1**	Upsample(2x2)<br>Conv2D(32, 3x3) + ReLU<br>BatchNorm<br>Conv2D(32, 3x3) + ReLU<br>BatchNorm	(8, 8, 32)
**Upsample Block 2**	Upsample(2x2)<br>Conv2D(16, 3x3) + ReLU<br>BatchNorm<br>Conv2D(16, 3x3) + ReLU<br>BatchNorm	(16, 16, 16)
**Upsample Block 3**	Upsample(2x2)<br>Conv2D(8, 3x3) + ReLU<br>BatchNorm<br>Conv2D(8, 3x3) + ReLU<br>BatchNorm	(32, 32, 8)
**Final Output**	Output Tensor	(32, 32, 8)

between the transformed representation and the attribute, $I(\phi_{\lambda,\delta}(\boldsymbol{z}_0), A)$. Since calculating MI directly is intractable for high-dimensional data, we instead optimize a tractable lower-bound using the Mutual Information Neural Estimation (MINE) framework [18]. This turns the problem into a min-max optimization game between the transformation function and a neural network that estimates the MI:

$$\min_{\lambda,\delta} \max_{\theta} \mathbb{E}[T_\theta(\phi_{\lambda,\delta}(\boldsymbol{z}_0), a)] - \log \left(\mathbb{E}_{\boldsymbol{Z}} \left[\mathbb{E}_A [e^{T_\theta(\phi_{\lambda,\delta}(\boldsymbol{z}_0), a)}] \right] \right) \tag{2}$$

where T_θ is the MINE estimator network. We solve this objective with an alternating optimization scheme. Below we describe the implementation of the two core components: the MINE estimator T_θ and the transformation $\phi_{\lambda,\delta}$.

Implementation of T_θ. The MINE framework relies on a neural network, which we denote T_θ, to provide a tight lower-bound on the mutual information. This network takes a (representation, attribute) pair as input and outputs a single scalar value.

- **Architecture:** The network T_θ uses a ResNet-18-style architecture. The latent representation $\boldsymbol{z}_0$ (shape: 32x32x8) is first concatenated with a one-hot encoding

Table 5. Network architecture for T_θ.

Layer/Block	Module	Output Shape
**Input**	Input Tensor = Concat($\boldsymbol{z}_0, Emb(a)$)	(32, 32, 10)
**Conv1**	Conv2D(10, 64, 3x3, stride=1, padding=1)<br>BatchNorm<br>ReLU	(32, 32, 64)
**Layer 1**	2 x Residual Block<br>(Conv2D(64, 64, 3x3, stride=1)<br>BatchNorm<br>ReLU)	(32, 32, 64)
**Layer 2**	2 x Residual Block<br>(Conv2D(64, 128, 3x3, stride=2)<br>BatchNorm<br>ReLU)	(16, 16, 128)
**Layer 3**	2 x Residual Block<br>(Conv2D(128, 256, 3x3, stride=2)<br>BatchNorm<br>ReLU)	(8, 8, 256)
**Layer 4**	2 x Residual Block<br>(Conv2D(256, 512, 3x3, stride=2)<br>BatchNorm<br>ReLU)	(4, 4, 512)
**Global Avg Pool**	AdaptiveAvgPool2d(1x1)	(1, 1, 512)
**Fully Connected**	Linear(512, 1)	(1, 1)
**Final Output**	Real Number Output	(1)

of the attribute a that has been tiled to match the spatial dimensions (shape: 32x32x2), resulting in an input tensor of shape 32x32x10. This tensor is processed through a series of residual blocks, followed by a global average pooling layer and a final fully connected layer to produce the scalar output. The full architecture is detailed in Table 5.

- **Training:** In the min-max optimization, the parameters θ of this network are updated to maximize the objective in Eq. (2). We use the Adam optimizer with a learning rate of $1e-4$. For every one update of the transformation network, we update the MINE estimator five times to ensure the MI estimate remains tight.

Implementation of the transformation function $\phi_{\lambda,\delta}$. The transformation ϕ is designed to minimally perturb the original latent representation $\boldsymbol{z}_0$ to remove the information about attribute A. The transformation is defined as:

$$\phi_{\lambda,\delta}(\boldsymbol{z}_0) = \boldsymbol{z}_0 + \lambda \frac{m_\delta(\boldsymbol{z}_0)}{\|m_\delta(\boldsymbol{z}_0)\|}$$

Here, m_δ is a neural network that predicts the optimal direction of perturbation in the latent space, and λ is a scalar hyperparameter that controls the magnitude of this perturbation.

- **Architecture:** The perturbation network m_δ must take a latent vector $\boldsymbol{z}_0$ and output a perturbation of the same dimension. We therefore employ a U-Net-like CNN architecture, which is well-suited for such image-to-image translation tasks. It consists of an encoder path that downsamples the representation and a decoder

path that upsamples it back to the original dimension, using skip connections to preserve fine-grained information. The full architecture is detailed in Table 4.

- **Training:** The parameters δ of the perturbation network are updated to *minimize* the MI estimated by T_θ. We again use the Adam optimizer with a learning rate of $1e-4$. The perturbation magnitude λ was set to $1e-3$ based on a random search.

4.3 Classifier for the self-reported race

The classifiers are instantiated with ResNet-101. Here we report the network architecture for better reproducibility. The network takes as input a grayscale image of size $256 \times 256 \times 1$. We adopt the implementation from torchvision package (torchvision.models.resnet101) with the first convolution layer modified to accept 1-channel inputs. After the final global average pooling layer, the 2048-dimensional vector is mapped by a fully connected layer with softmax activation. The optimization uses Adam optimizer with 1e-4 learning rate. The total training epoch number is 30 and the batch size is 15.

References

1. Bongers S, Forré P, Peters J, Mooij JM. Foundations of structural causal models with cycles and latent variables. The Annals of Statistics. 2021;49(5):2885 – 2915.
2. Pearl J. Causality: Models, Reasoning and Inference. Cambridge University Press; 2000.
3. Pearl J. Causality: Models, Reasoning and Inference. Cambridge University Press; 2009.
4. Peters J, Janzing D, Schlkopf B. Elements of Causal Inference: Foundations and Learning Algorithms. The MIT Press; 2017.
5. Williams D. Probability with Martingales. Cambridge University Press; 1991.
6. Irvin J, Rajpurkar P, Ko M, Yu Y, Ciurea-Ilcus S, Chute C, et al. CheXpert: A Large Chest Radiograph Dataset with Uncertainty Labels and Expert Comparison. In: The Thirty-Third AAAI Conference on Artificial Intelligence, AAAI 2019, The Thirty-First Innovative Applications of Artificial Intelligence Conference, IAAI 2019, The Ninth AAAI Symposium on Educational Advances in Artificial Intelligence, EAAI 2019, Honolulu, Hawaii, USA, January 27 - February 1, 2019. AAAI Press; 2019. p. 590–597.
7. Yuan Z, Yan Y, Sonka M, Yang T. Large-scale Robust Deep AUC Maximization: A New Surrogate Loss and Empirical Studies on Medical Image Classification. In: 2021 IEEE/CVF International Conference on Computer Vision, ICCV 2021, Montreal, QC, Canada, October 10-17, 2021. IEEE; 2021. p. 3020–3029.
8. Johnson AEW, Pollard TJ, Berkowitz SJ, Greenbaum NR, Lungren MP, ying Deng C, et al. MIMIC-CXR, a de-identified publicly available database of chest radiographs with free-text reports. Scientific Data. 2019;6.
9. Johnson AEW, Pollard TJ, Greenbaum NR, Lungren MP, ying Deng C, Peng Y, et al.. MIMIC-CXR-JPG, a large publicly available database of labeled chest radiographs; 2019.

10. Huang G, Liu Z, Weinberger KQ. Densely Connected Convolutional Networks. CoRR. 2016;abs/1608.06993.

11. Simonyan K, Zisserman A. Very Deep Convolutional Networks for Large-Scale Image Recognition; 2015.

12. Szegedy C, Vanhoucke V, Ioffe S, Shlens J, Wojna Z. Rethinking the Inception Architecture for Computer Vision; 2015.

13. Dhariwal P, Nichol A. Diffusion models beat gans on image synthesis. NeurIPS. 2021; p. 8780–8794.

14. Müller-Franzes G, Niehues JM, Khader F, Arasteh ST, Haarburger C, Kuhl C, et al. A multimodal comparison of latent denoising diffusion probabilistic models and generative adversarial networks for medical image synthesis. Scientific Reports. 2023;13(1).

15. Ronneberger O, Fischer P, Brox T. U-Net: Convolutional Networks for Biomedical Image Segmentation. CoRR. 2015;abs/1505.04597.

16. Song J, Meng C, Ermon S. Denoising Diffusion Implicit Models. CoRR. 2020;abs/2010.02502.

17. Nichol A, Dhariwal P. Improved Denoising Diffusion Probabilistic Models. CoRR. 2021;abs/2102.09672.

18. Belghazi MI, Baratin A, Rajeswar S, Ozair S, Bengio Y, Hjelm RD, et al. Mutual Information Neural Estimation. In: Proceedings of the 35th International Conference on Machine Learning, ICML 2018, Stockholmsmässan, Stockholm, Sweden, July 10-15, 2018. vol. 80 of Proceedings of Machine Learning Research. PMLR; 2018. p. 530–539.
